# How pathological criteria can impact prognosis of tongue and floor of the mouth squamous cell carcinoma

**DOI:** 10.1590/1678-7757-2019-0198

**Published:** 2019-11-15

**Authors:** Renata Miranda Rodrigues, Vagner Gonçalves Bernardo, Sabrina Daniela Da Silva, Danielle Resende Camisasca, Paulo Antônio de Silvestre Faria, Fernando Luiz Dias, Luís Felipe Ribeiro Pinto, Rodolpho Mattos Albano, Anke Bergmann, Simone de Queiroz Chaves Lourenço

**Affiliations:** 1 Universidade Federal Fluminense, Programa de Graduação em Odontologia, Niterói, Rio de Janeiro, Brasil.; 2 Universidade Estadual do Rio de Janeiro (UERJ), Departamento de Bioquímica, Rio de Janeiro, Rio de Janeiro, Brasil.; 3 Segal Cancer Centre, Department of Otolaryngology, Head and Neck Surgery; Sir Mortimer B. Davis-Jewish General Hospital, Lady Davis Institute for Medical Research, Montréal, Canada.; 4 Universidade Federal do Espírito Santo, Departamento de Patologia Oral, Vitória, Espírito Santo, Brasil.; 5 Instituto Nacional de Câncer (INCA), Divisão de Patologia, Rio de Janeiro, Rio de Janeiro, Brasil.; 6 Instituto Nacional de Câncer (INCA), Departamento de Cirurgia de Cabeça e Pescoço, Rio de Janeiro, Rio de Janeiro, Brasil.; 7 Instituto Nacional de Câncer (INCA), Centro de Pesquisas, Programa de Carcinogênese Molecular.

**Keywords:** Oral cancer, Squamous cell carcinoma, Prognosis, Survival, Oral pathology

## Abstract

Pathological parameters have been indicated as tumor prognostic factors in oral carcinoma.

**Objective:**

The objective of this study was to investigate the impact of pathological parameters on prognosis of patients affected only by tongue and/or floor of the mouth squamous cell carcinoma (SCC).

**Methodology:**

In total, 380 patients treated in the Brazilian National Cancer Institute (INCA) from 1999 to 2006 were included. These patients underwent radical resection followed by neck dissection. The clinical and pathological characteristics were recorded. The Kaplan-Meier method and Cox proportional hazards model were used in survival analysis. Overall survival (OS), cancer-specific survival (CSS) and disease-free interval (DFI) were estimated. Cox residuals were evaluated using the R software version 3.5.2. Worst OS, CSS and DFI were observed in patients with tumors in advanced pathological stages (p<0.001), with the presence of perineural invasion (p<0.001) and vascular invasion (p=0.005).

**Results:**

Advanced pathological stage and the presence of a poorly differentiated tumor were independent prognostic factors for OS and CSS. However, advanced pathological stage and perineural invasion were independent predictors of a shorter OS, DFI and CSS.

**Conclusion:**

Pathological stage and perineural invasion were the most significant pathological variables in survival analysis in tongue and/or floor of the mouth SCC.

## Introduction

Head and neck cancer is the sixth most common type of cancer,^1^ being strongly associated with tobacco and alcohol consumption. Several regions, including the oral cavity, can be affected by these tumors. Squamous cell carcinomas (SCC) are the most common type of tumor in this region. Oral cancer is usually caused by epithelial lining cell transformation. The incidence of 354,864 new cases for both sexes was expected worldwide in 2018.^1^ When compared to other South American countries, Brazil presented the highest rates of oral cancer.^2^ Data from the Brazilian National Cancer Institute (INCA) estimated that oral cancer was the fifth most common type of cancer among men and the 12^th^ among women in 2018.^3^

Tongue is the most commonly affected site in oral cavity, followed by the floor of the mouth.^4^ Studies show that tumors on this site have a worse prognosis than other sites in the oral cavity due to their incomplete response to treatment strategies, resulting in lower survival rates.^5^ Consequently, cancer of the oral cavity has higher mortality rates (50%) when compared to other types of cancer.^1^

The TNM classification is used clinically to define therapy and estimate its response and prognosis.^6^ However, it does not necessarily reflect a precise impact on prognosis. Tumors associated with the same stage of this classification can show different disease progression.^7^^–^^8^ New parameters such as depth of invasion and extracapsular extravasation, were included in the 8^th^ edition of TNM (2017) to improve their predictive value and different stages stratification, and to reorganize patients previously considered low risk but had a shorter survival.

The pathological parameters described as tumor prognostic indicators in oral SCC are: pathological stage, histopathological grading of the World Health Organization (WHO),^9^ presence of vascular and perineural invasion, extracapsular spread and positive surgical margins.^9^^–^^12^

Therefore, this study sought to investigate the impact of pathological parameters on the prognosis of patients with tumors in tongue and floor of the mouth SCC only, and treated primarily by radical surgery in a single institution (INCA).

## Methodology

### Study population

Data from 380 patients with oral SCC diagnosed between 1999 and 2006 were extracted from a database of the Department of Head and Neck Surgery (Process #125/10 – INCA ethics committee). The tumors of these patients affected only oral tongue and the floor of the mouth. The exclusion criteria were: being a second primary cancer, presence of distant metastasis at the time of diagnosis and with a follow-up that lasted less than one month. All patients underwent radical resection as initial treatment and had surgical margins free of tumor, confirmed either with frozen section or with final pathological examination. The first treatment for the primary carcinoma also included neck dissection. In total, 204 patients received postoperative therapy (radiotherapy). Chemotherapy was not indicated for any patient.

### Data collection

All medical records and pathologic reports were reviewed and the data were collected as shown in Table 1. Pathological data included tumor stage (pTNM). The 7^th^ edition of TNM Classification of Malignant Tumors (UICC staging system) was used to classify the tumors as either early (I/II) or advanced (III/IV),^13^ the presence of vascular permeation and perineural invasion, and tumor differentiation, according to the WHO grading system.^9^

**Table 1:** Clinicopathological features of the samples (n=380)

Variable	Category	No. of cases	% of cases
Gender	Male	292	76.8
	Female	88	23.2
Age	<40 years	28	7.4
	≥40 <65 years	255	67.1
	≥65 years	97	25.5
Tobacco	No	56	14.7
	Current or Past	322	84.7
	Not recorded	2	0.6
Alcohol	No	73	19.2
	Current or Past	304	80.0
	Not recorded	3	0.8
Tumor site	Tongue	210	55.3
	Floor of mouth	78	20.5
	Both	92	24.2
Clinical stage	I	84	22.1
	II	160	42.1
	III	99	26.1
	IV	37	9.7
Pathological stage	I	77	20.3
	II	99	26.1
	III	82	21.6
	IV	122	32.0
pN	N0	206	54.2
	N1+2	174	45.8
Grading	Well	67	17.6
	Moderately	288	75.8
	Poorly	18	4.7
	Not recorded	7	1.9
Perineural invasion	No	293	77.1
	Yes	87	22.9
Vascular invasion	No	332	87.4
	Yes	48	12.6
Extracapsular spread	No	354	93.2
	Yes	26	6.8
Adjuvant treatment	No	176	46.3
	Yes	204	53.7
Disease progression	No	309	81.3
	Yes	71	18.7
Second primary tumor	No	321	84.5
	Yes	59	15.5

### Statistical analysis

For descriptive and statistical analysis, the SPSS statistics package for Windows (version 20.0, IBM Corp., Armonk, NY, USA) was used. Analysis of survival was estimated using surgery date as starting point. The overall survival (OS), cancer-specific survival (CSS) and disease-free interval (DFI) were estimated using the Kaplan-Meier method and log-rank test for curve comparison. To evaluate prognostic factors associated with pathological parameters, hazard ratios (HR) were estimated with 95% confidence intervals (95%CI). The Cox proportional hazards model was used in each group after controlling for potentially confounding variables. Cox residuals were evaluated using the R software version 3.5.2. The tests were considered statistically significant when the p-value was <0.05.

## Results

All the 380 patients of a single institution received the same protocol procedures for diagnosis and treatment. Tumor-derived specimens had the same histological type (squamous cell carcinoma) and site (tongue and floor of the mouth). The profile of the studied population followed REMARK's recommendations.^14^^,^^15^

### Clinicopathological results

Clinical and pathological characteristics are presented in Table 1. In total, 380 patients, 292 males (76.8%) and 88 females (23.2%), with 57 years as the median age were enrolled in this study (range 20–93 years). Most cases were tongue SCCs (n=210, 55.3%). In total, 78 tumors (20.5%) were floor of the mouth SCCs and 92 tumors (24.2%) affected both sites, simultaneously. Most patients showed clinical stage II (n=160; 42.1%), followed by stage III (n=99; 26.1%). Pathologic staging was: stage IV (n=122; 32.1%); stage II (n=99; 26.1%); stage III (n=82; 21.6%) and stage I (n=77; 20.3%). Lymph node (LN) neck dissection was performed in all patients (259 unilateral lymph node dissections and 121 bilateral lymph node dissections). Pathological lymph node metastasis (pN) was detected in 174 patients (45.8%). Surgical treatment was indicated as the only treatment for 176 patients (46.4%).

The median follow-up period was 4.3 years (range 0.2–10 years). The overall recurrence rate was 42.6% (n=150) including local recurrences, cervical metastases and distant metastases. A second primary tumor was found in 59 cases (15.5%). Clinical and pathological characteristics are presented in Table 1.

### Survival analysis

**Figure 1:**
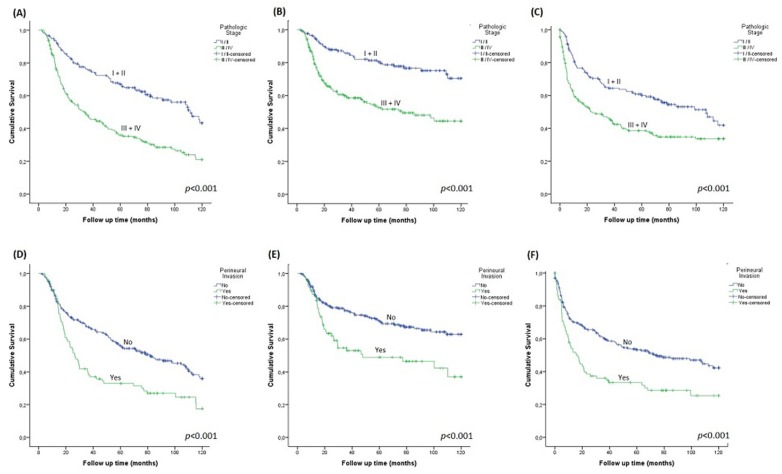
Survival charts of pathological stages. A- Overall (OS); B- Cancer-specific survival (CSS); C- Disease-free interval (DFI) and perineural invasion; D- OS; E- CSS; F- DFI

The 10-year OS, CSS and DFI rates were 55.3%, 33.2% and 40.8%, respectively. Kaplan-Meier curves revealed that patients with advanced pathologic stage tumors (III and IV) showed worse OS (95%CI 46.5–59.4; p<0.001), CSS (95%CI 62.2–77.3; p<0.001) and DFI (95%CI 44.2–59.1; p=0.002) (Figure 1A, B, C). Tumors with perineural invasion showed worse OS (95%CI 40.2–59.2; p<0.001), CSS (95%CI 54.8–77.5; p<0.001) and DFI (95%CI 33.0–54.3; p<0.001) (Figure 1D, E, F).

**Figure 2:**
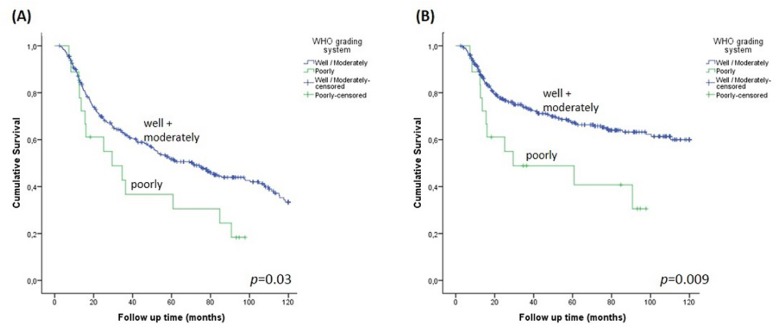
Survival charts of WHO grading system. A- Overall (OS); B- Cancer-specific survival (CSS)

Poorly differentiated tumors (WHO system) had prognostic value for both OS (p=0.03) and CSS (p=0.009) (Figure 2). Significantly, lower DFI values were observed in the presence of perineural and vascular invasion and pathological stage III/IV (Table 2).

**Table 2:** Pathological characteristics and survival: univariate analysis

		Overall survival		Cancer-specific survival		Disease-free interval	
Variable	No. of cases	(95% CI)	p value	(95% CI)	p value	(95% CI)	p value
Pathological Stage							
I/II	176	(76.7 – 90.2)		(92.2 – 104.6)		(66.9 – 82.4)	
III/IV	204	(46.5 – 59.4)	<0.001	(62.2 – 77.3)	<0.001	(44.2 – 59.1)	<0.001
Grading system							
Well/ Moderately	355	(63.7 – 73.9)		(80.1 – 90.6)		(57.3 – 68.7)	
Poorly	18	(28.3 – 61.2)	0.03	(33.8 – 70.4)	0.009	(21.9 – 71.3)	0.23
Perineural invasion							
Yes	87	(40.2 – 59.2)		(54.8 – 77.5)		(33.0 – 54.3)	
No	293	(67.1 – 78.2)	<0.001	(82.9 – 94.1)	<0.001	(61.9 – 74.4)	<0.001
Vascular invasion							
Yes	48	(33.4 – 56.8)		(46.9 – 76.2)		(30.7 – 58.5)	
No	332	(65.5 – 76.0)	<0.001	(81.2 – 92.0)	<0.001	(59.3– 71.1)	0.005
Extracapsular spread							
Yes	26	(62.0 – 99.8)		(81.6 – 115.4)		(51.6 – 92.4)	
No	354	(61.0 – 71.1)	0.09	(76.8 – 87.5)	0.10	(55.9 – 67.3)	0.32

### Multivariate Cox regression analysis

The variables were analyzed by the Kaplan-Meier method (univariate analysis) and, when p-values were ≤0.20, they were included in the Cox regression model for multivariate analysis (log-rank test). Multivariate analysis showed pathological stage III/IV (HR=2.1; 95%CI 1.60–2.87; p<0.001) and perineural invasion (HR=1.47; 95%CI 1.08–1.99; p=0.01) as independent factors for a lower OS (Table 3).

**Table 3:** Multiple regression Cox analysis for the factors influencing overall, cancer-specific and disease-free interval

Variable	Category	Overall survival		Cancer-specific survival		Disease-free interval	
		HR (95% CI)	p Value	HR (95% CI)	p Value	HR (95% CI)	p Value
Pathological Stage	I+II/ III+IV	2.14 (1.60 – 2.87)	**<0.001**	2.72 (1.81 – 4.09)	**<0.001**	1.64 (1.22 – 2.21)	**<0.001**
Perineural invasion	Yes/ No	1.47 (1.08 – 1.99)	**0.01**	1.57 (1.06 – 2.31)	**0.02**	1.58 (1.15 – 2.16)	**0.004**
Grading system	Well+ Moderately/ Poorly	a	a	2.0 (1.07 – 3.73)	**0.03**	a	a

a Variable not included in the analysis

Likewise, pathological stage III/IV (HR=2.72; 95%CI 1.81–4.09; p<0.001), perineural invasion (HR=1.57; 95%CI 1.06–2.31; p=0.02) and poorly differentiated tumors (HR=2.00; 95%CI 1.07–3.73; p=0.03) were significant for CSS.

The independent predictive factor for a lower DFI was pathological stage III/IV (HR=1.64; 95%CI 1.22–2.21; p<0.001) and perineural invasion (HR=1.58; 95%CI 1.15–2.16; p=0.004) (Table 3).

## Discussion

Oral SCC remains as one of the most difficult malignancies to control due to its high tendency for local invasion and cervical lymph node dissemination, especially the tongue and floor of the mouth SCC.^16^^,^^17^ This is the reason why its survival rates remain low, even with advances in therapeutic strategies.^18^ The prediction of tumor behavior is difficult when using only conventional clinical and histological parameters.^19^

Several studies include reliable pathological parameters in oral tumor staging to improve the prognosis.^7^^–^^20^^,^^21^ Some studies investigated the prognostic role of pathologic parameters in oral SCC, in general, analyzing the parameters separately.^11^^–^^24^ On the other hand, our study comprised most of the pathological criteria, including pathological stage, WHO grading system, perineural infiltration and vascular invasion.

The 8^th^ edition of TNM Classification of Malignant Tumours, proposed in 2017,^12^ is a strong independent predictor factor of both OS and CSS for SCC in tongue and floor of the mouth. By including extracapsular extension and depth of invasion as critical prognostic factors for SCC of the oral cavity, it may effectively measure the risk associated with pathological variables. Thus, an increase in the depth of invasion probably precedes and influences perineural and perivascular invasions. In addition, perineural invasion and WHO grading system could be included in TNM classification for a more complete variable, because these are the most predictable variables, as suggested by Subramaniam, et al.^25^ (2019), using the new edition of TNM, and also our study, using the 7^th^ edition.

We were unable to assess the depth of invasion in our study due to only using medical records. Also, this study was carried out before the publication of the new edition of the TNM Classification of Malignant Tumours.^12^ Therefore, we are planning to include these new parameters in the studied population.

Moreover, we selected, according to our inclusion criteria, patients considered free surgical margins. Positive margins can significantly decrease overall survival and increase the risk of local recurrence.^9^^,^^26^

Furthermore, our data suggest that pathological TNM staging is an important prognostic factor due to its ability to predict not only OS and CSS, but also DFI. In addition, this variable predicts both OS and CSS independently. Amit, et al.^27^ (2018) showed the same results, with a significant association with OS and CSS in multivariate analysis for pathological TNM stage.

Our study confirms that perineural invasion is a predictor of unfavorable DFI, which was already observed in a previous study with the same criteria.^24^ Perineural invasion was also a predictor of worst OS and CSS and showed an independent prognostic value in multivariate analysis. Vascular invasion has been significantly associated with survival^11^, but our study also showed it as a significant prognostic indicator for OS, CSS and DFI.

Also, our data showed poor differentiated tumors as an independent predictor for CSS. WHO grading system was a predictor of worst CSS in multivariate analysis, thus emerging as an important pathologic parameter. Poorly differentiated tumors presented higher recurrence rates and showed a shorter cancer specific survival when compared to well and moderately differentiated tumors. Although some authors did not prove its independent prognostic value,^28^^,^^29^ other authors had the same conclusion, as reported by Lindenblatt, et al.^30^ (2012) and Sopka, et al.^31^ (2013). These conclusions reflect that the biology of undifferentiated tumors is usually associated with increased mitotic activity and invasiveness. We propose further studies of this variable to confirm our findings, and perhaps allow its inclusion in the TNM Classification.

As limitations of this study we point the use of secondary sources, such as histopathological reports and medical records, and the unavailability of some information. Histopathological reports were performed by different pathologists from the same institution. We suggest an additional study including tumors in other sites to complement our observations. However, our findings reinforce the need to describe all pathologic parameters in histopathological reports.

## Conclusion

In conclusion, pathological criteria have direct impact in patient's prognosis. In addition, an independent predictor of survival was found for pTNM, perineural invasion and WHO grading system. Our study highlights the fact that the WHO grading system is also a promising prognostic indicator for tongue and floor of the mouth SCC.
